# Understanding Lesion Creation Biophysics and Improved Lesion Assessment during Radiofrequency Catheter Ablation. The Perfect Combination to Achieve Durable Lesions in Atrial Fibrillation Ablation

**DOI:** 10.31083/j.rcm2502044

**Published:** 2024-01-29

**Authors:** Ely Gracia, Andres F. Miranda-Arboleda, Carolina Hoyos, Carlos D. Matos, Jose Osorio, Jorge E. Romero, Paul C. Zei

**Affiliations:** ^1^Cardiac Arrhythmia Service, Cardiovascular Division, Brigham and Women’s Hospital, Harvard Medical School, Boston, MA 02115, USA; ^2^HCA Electrophysiology, Mercy Hospital, Miami, FL 33133, USA

**Keywords:** atrial fibrillation, pulmonary vein isolation, biophysics, lesion assessment, impedance, contact force, ablation index

## Abstract

Atrial fibrillation (AF) is a prevalent arrhythmia, while pulmonary vein isolation (PVI) has become a cornerstone in its treatment. The creation of durable lesions is crucial for successful and long-lasting PVI, as inconsistent lesions lead to reconnections and recurrence after ablation. Various approaches have been developed to assess lesion quality and transmurality *in vivo*, acting as surrogates for improved lesion creation and long-term outcomes utilizing radiofrequency (RF) energy. This review manuscript examines the biophysics of lesion creation and different lesion assessment techniques that can be used daily in the electrophysiology laboratory when utilizing RF energy. These methods provide valuable insights into lesion effectiveness, facilitating optimized ablation procedures and reducing atrial arrhythmia recurrences. However, each approach has its limitations, and a combination of techniques is recommended for comprehensive lesion assessment during AF catheter ablation. Future advancements in imaging techniques, such as magnetic Resonance Imaging (MRI), optical coherence tomography, and photoacoustic imaging, hold promise in further enhancing lesion evaluation and guiding treatment strategies.

## 1. Introduction

Pulmonary vein isolation (PVI) utilizing thermal ablation has become one of the most effective and widely employed ablation modalities for the management of atrial fibrillation (AF) [1, 2]. Thermal ablation lesion creation can be achieved with either radiofrequency (RF) energy application or cryoablation application, each offers unique mechanisms for generating tissue injuries. Application of RF ablation energy results in direct cellular lysis and immediate necrosis. Cryoablation results in irreversible alterations to the cytoplasmic components of cells without destroying the cellular membrane [3].

Restoration and maintenance of sinus rhythm via catheter ablation have been associated with the remodeling of the left atrium. Several studies have demonstrated that following the restoration of the sinus rhythm via catheter ablation, there is a significant reduction in the left atrial dimension as well as the geometry of the pulmonary vein ostia [4, 5].

As the cornerstone of AF ablation, the objective of radiofrequency ablation (RFA) is to create continuous, transmural lesions, utilizing sufficient energy delivery that results in irreversible electrical isolation and permanent cellular damage, without subjecting surrounding structures to collateral damage [6, 7, 8, 9]. Inconsistent lesions have been associated with reconnections and AF recurrence after ablation [10, 11].

The purpose of this review is to identify the key aspects in the biophysics of lesion creation and review the different available tools to assess transmural and effective RFA lesions.

## 2. Biophysics of Radiofrequency Lesion Creation 

**Fig. 1.**
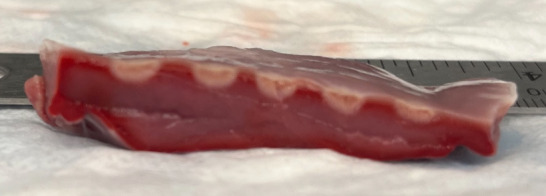
**Typical lesion created using high power–short duration set up at 50 W power, 10 g force, and 5 seconds duration**. This picture illustrates lesion depth, morphology, diameter, and differentiation in resistive and conductive heating areas.

Traditionally, RF lesion formation has relied on the application of moderate power, ranging from 25 to 35 Watts, delivered over a maximum period of 60 seconds, while maintaining contact forces (CFs) between 10 and 20 g. However, this ablation strategy was associated with high rates of pulmonary vein reconnections at 3 months post PVI, although these were thought to be secondary to catheter instability and tissue edema, which eventually failed to create a permanent lesion [1, 6]. To address these elevated recurrence rates, while minimizing the risk of thermal injury to surrounding tissues, an ablation strategy employing the delivery of high wattage (40–50 W) over a short period, commonly referred to as a high power–short duration (HPSD) ablation approach, has been developed and has resulted in improved freedom from AF at one year with no increase in collateral damage to the adjacent structures (Fig. 1, Fig. 2.1) [12].

**Fig. 2.**
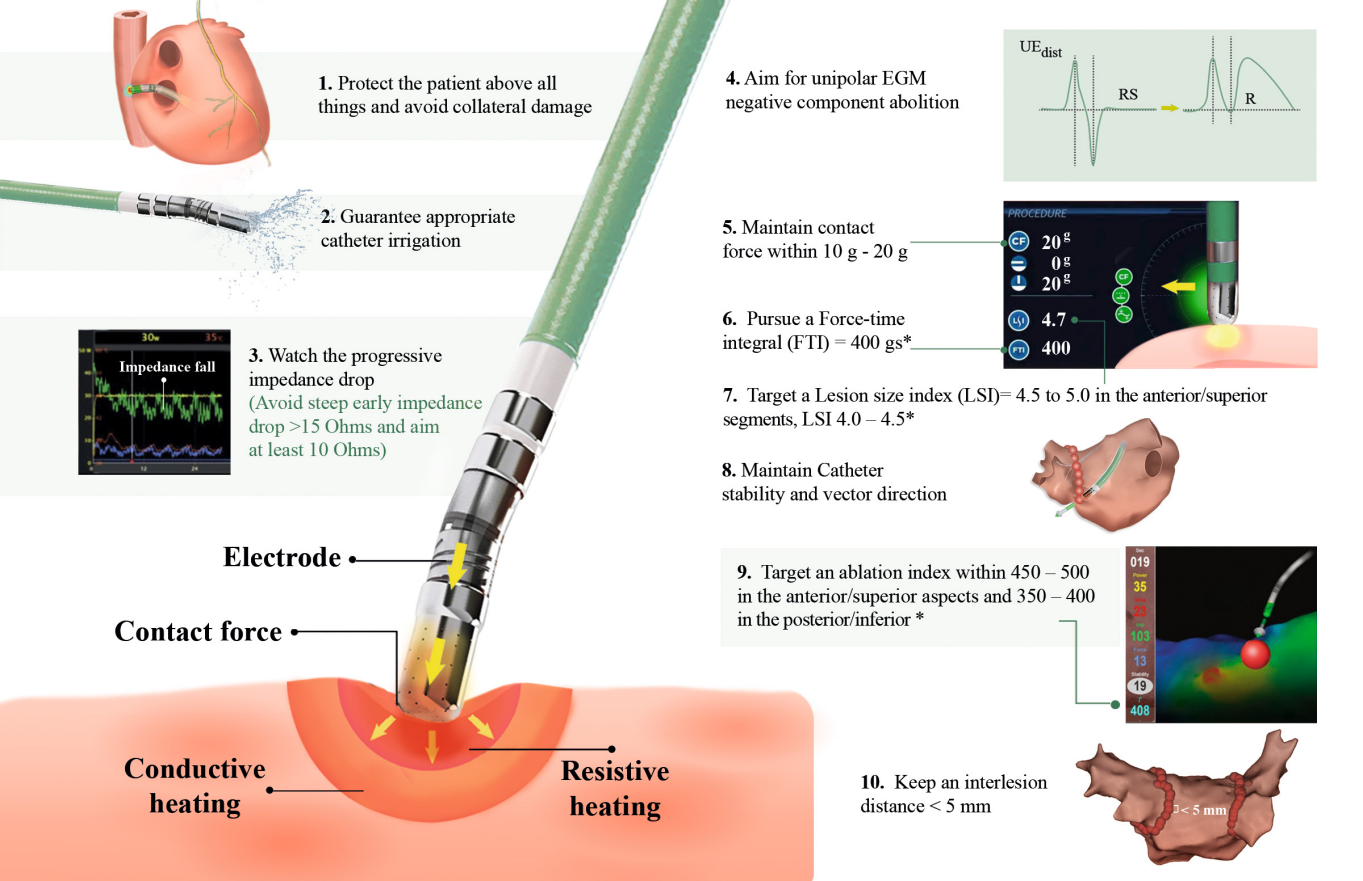
**Ten commandments for durable lesion creation and assessment based on available surrogates of tissue transmurality**. * Stop ablation earlier if signs of collateral damage are present: elevation of esophageal temperature, late progressive impedance rise during ablation, and early steep impedance decrease during ablation. EGM, electrogram.

Lesions created using RF energy rely on a thermal injury occurring in two consecutive heating phases. The initial heating phase, known as resistive heating, occurs immediately on the tissue–catheter interphase, where electric current is transmitted directly onto the superficial tissue layer. During the subsequent phase, passive conductive heating propagates through the tissue, resulting in a deeper lesion formation (Fig. 1) [13]. Both resistive and conductive phases are time-dependent, with shorter lesion applications relying on resistive heating and longer applications relying on conductive heating [6, 13].

In addition to the power and duration of RF application, there exist other variables that can be manipulated, which ultimately impact lesion creation, including irrigation and fluid tonicity (Fig. 2.2). Non-irrigated tip ablation catheters rely on convective cooling at the tissue–catheter interface and result in the creation of larger diameter, hemisphere-shaped lesions closer to the tissue surface [14]. Non-irrigated ablation catheters reach the set temperature limits more quickly, effectively reducing the amount of current that can be delivered into the tissue and limiting the propagation of heat deep into the tissue. Open-irrigated catheters, which utilize saline to cool the tissue–catheter interphase, allow for longer application durations at a higher power of delivery at the tissue-catheter interphase, resulting in a teardrop-shaped lesion with a deeper maximal width, compared to lesions created by non-irrigated catheters [14, 15, 16]. Additionally, by immediately reducing the temperature at the tissue–catheter interphase, the lesions created with irrigated catheters reduce the rates of char and thrombus formation [17].

The durability of lesion formation is crucial in providing persistent pulmonary vein isolation. With RF energy delivery, there is cellular membrane destabilization, edema, and eventual cellular necrosis; however, many factors make achieving this goal challenging. Prior studies have shown that there is a significant amount of tissue edema that results from the application of RF energy onto tissue and this edema may contribute to a transient, reversible block [3]. A potential way to avoid the effects of acute tissue edema is to optimize certain aspects of lesion formation, including catheter stability, to achieve the appropriate contact forces and adjustments in power delivery in order to generate durable lesions.

## 3. Ablation Lesion Assessments. Are We Using the Appropriate Tools? 

Histopathology is considered the gold standard for experimentally assessing the effectiveness of lesions delivered during ablation [7]. However, obtaining real-time histopathological information during the procedure is of course not feasible. Therefore, various tools have been developed to assess the quality and transmurality of lesions *in vivo*, serving as surrogates for better lesion creation and, consequently, improved mid and long-term outcomes after catheter ablation (Table 1, Ref. [18, 19, 20, 21, 22, 23, 24]) [8].

**Table 1.** **Comparison of outcomes after radiofrequency catheter ablation for AF using different lesion assessment parameters**.

Variable	Impedance modification [18]	EGM changes [19]	Contact force [20]	FTI [21]	LSI [22]	Alation index and interlesion distance [23]	Electrical excitability [24]
Targeted parameter	Impedance drops (14 ± 8 Ohms vs. 6 ± 4)	Ablation is delivered until unipolar EGM becomes monophasic positive	Average force 26.8 ± 10.7	CFs between 10 to 20 g and an FTI of 400 gs	LSI of 6.0 in the left pulmonary veins ridge, 5.5 in the anterior, and 5.0 in the posterior segments of the PVs	AI of at least 400 in the posterior wall and 550 in the anterior wall and interlesion distance <6 mm	Loss of capture along the line after PVI
Freedom from atrial arrhythmias after 1-year follow-up	82%	85%	84%	85%	86%	94%	83%
Mean follow-up	366 ± 130 days	12 months	12 months	3 months	24 months	12 months	24 months

AI, ablation index; EGM, electrogram; FTI, force-time integral; LSI, lesion size integral; PVI, pulmonary vein isolation; AF, atrial fibrillation; CFs, contact forces; PVs, pulmonary veins.

### 3.1 Impedance Modification Variables

Early studies demonstrated that impedance is a dynamic parameter during catheter ablation. Harvey *et al*. (1992) [25] showed that a 10 Ohms impedance decrease during the ablation of accessory pathways or the atrioventricular (AV) node junction predicted tissue heating through abrupt conduction interruption. Moreover, sudden increments in impedance were associated with thrombus formation at the catheter tip. Animal studies have also revealed that sudden increases in impedance during ablation are associated with significant electrode–tissue interphase temperatures, exceeding 100 °C, resulting in tissue denaturation, boiling, creation of steam pops, and clot formation [26].

Impedance changes during ablation can be attributed to a progressive increase in tissue temperature, which enhances the mobility of ions in the solution, leading to a decrease in the resistance to the current flow [27]. A significant and early decrease in impedance is associated with a higher risk of tissue damage and steam pop formation. A sudden impedance drop of more than 15 Ohms during the first 2 seconds of ablation predicts the subsequent significant impedance rise. Conversely, lesions not terminating in a sudden increase exhibited an initial impedance drop of 3.2 Ohms during the first two seconds of catheter energy delivery (Fig. 2.3) [27].

Prior studies have confirmed that ablation lesions with impedance drops of less than 10 Ohms during an index PVI procedure were present in 89% of the areas with conduction recovery during a re-do intervention. The most common areas of reconnection were found in the posterior antra [28]. De Bortoli *et al*. [29] established a correlation between impedance reduction during ablation and CF, demonstrating that a CF greater than 5 g produced a better impedance decrease. However, CFs exceeding 20 g were related to impedance increments at the end of the ablation and tissue overheating [29].

Impedance is measured with radiofrequency generators from the tip of the ablation catheter to an indifferent electrode placed on the patient’s skin. This measurement is susceptible to influences from various factors, including abnormalities in the chest wall, muscles, obesity, and alterations in the patch–patient interface (e.g., sweat, air), which limit the use of circuit impedance as a reliable measurement [30].

Advancements have allowed the development of techniques that calculate local tissue impedance using mini electrodes located in the catheter tip. Clinical studies have concluded that assessing local impedance during ablation can differentiate local myocardium from the blood pool, provide information about the catheter orientation, and tissue thickness, and indicate lesion dimension. Furthermore, accelerated local impedance drops may be associated with a higher risk of steam pops [30].

The LOCALIZE trial (Local catheter impedance drop during pulmonary vein isolation), which utilized a new local impedance-based catheter, involved performing mapping procedures three months after the first PVI to assess the characteristics of durable lesions [31]. The study found that a local impedance drop was a better predictor of a durable conduction block compared to a generator impedance. The optimal delta local impedance changes in the left atrium (LA) were 16.8 Ohms in the anterior/superior areas and 14.2 Ohms in the posterior/inferior, with positive predictive values for a durable conduction block of 97.7% and 96.9%, respectively. Baseline local impedance was found to be different in healthy tissues, gaps, and established scars. An optimal baseline impedance of 110 Ohms was determined to achieve greater local impedance drops [31]. Results from the CHARISMA (Catheter Ablation of Arrhythmias with a High-Density Mapping System in Real-World Practice) registry showed that successful ablation lesions had greater local impedance drops (14 ± 8 Ohms vs. 6 ± 4) and demonstrated that the rate of atrial arrhythmia recurrence was 18% after a mean follow-up of 366 ± 130 days [18].

While impedance drops offer valuable insights, they have limitations as predictors of transmural lesions. Their utility can only be used after ablation has started, and there is no clear impedance drop cutoff and corresponding time that accurately predicts transmurality. As demonstrated before, most of the data using baseline impedance or impedance variation comes from paroxysmal AF patients, meaning these variables have not been widely studied in patients with persistent AF, LA fibrosis, or prior ablation procedures, which may limit their use in this subset of patients. Additionally, generator-based impedance measurements are affected by other factors, such as indifferent electrode position, hemodynamic conditions, body composition, and generator connections.

### 3.2 Changes in Electrogram Morphology during Ablation: A Parameter for Lesion Transmurality

Assessing the change in electrogram (EGM) morphology during ablation has emerged as an alternative parameter to define lesion transmurality in the treatment of AF [32, 33, 34, 35]. Experimental studies in animals have demonstrated that achieving an 80% reduction in unipolar EGMs is associated with the development of transmural, long-lasting lesions after the initial ablation [36]. However, the EGM modification was smaller and had a higher incidence of non-transmural lesions in the trabeculated areas of the LA [35]. Alternatively, the elimination of the negative component on unipolar EGMs was found to be associated with transmural lesions, independently of the catheter orientation [34]. Modifications to the unipolar electrogram characteristics have been shown to provide more relevant information. In bipolar recordings, the signals from the ring electrode tend to dominate the EGM and lead to a potential rise in bipolar amplitude after ablation owing to a greater signal difference between both electrodes [32, 37]. In bipolar EGMs, the signs of transmurality included the elimination of a positive deflection with a non-parallel catheter orientation and the attenuation of an existent R wave, which was higher than 75% in areas with QRS morphology EGM patterns, or the complete elimination of an R’ wave in areas with a preablation RSR’ morphology [34].

Unipolar signal modification has been used as a guide to define lesion creation in patients undergoing PVI to treat AF. RF applications were delivered until the unipolar EGM had a total abolition of the negative component and turned completely positive. Compared to empiric 30-second RF applications, the unipolar EGM approach was associated with a lower recurrence rate of atrial arrhythmias after 21 ± 4 months (88% vs. 70%, respectively) [38].

Clinical studies have demonstrated that the time to achieve a monophasic R wave unipolar EGM was less than 7 seconds when a power of 30 W in the LA posterior wall and a CF between 11 g and 16.5 g was used [39]. The results of the UNIFORCE study (Elimination of the negative component of the unipolarelectrogram as a local procedural endpoint during paroxysmalatrial fibrillation catheter ablation using contact-force sensing: the UNIFORCE study) showed that an ablation approach that targeted the elimination of the negative component of the unipolar signal during the application of RF energy in patients with paroxysmal AF undergoing PVI resulted in a long-lasting effect. After a two-year follow-up, 87% of the patients remained free of arrhythmia without administering antiarrhythmic drugs (Fig. 2.4) [40].

A prospective multicenter randomized study compared a catheter ablation, guided by a unipolar signal modification, to CFs, in the unipolar signal group, an ablation was delivered until a completely positive EGM was developed. After a 12-month follow-up, there was a significant difference in the time the patients were free of atrial arrhythmias in both groups, with 85% of patients free in the EGM group vs. 70% in the CF group [19].

Contradictory results were found in another randomized controlled trial comparing the administering of an EGM-guided approach until the complete abolition of the negative component in the unipolar signals vs. an LSI-guided approach (4.5–5.0 in the anterior/superior segments and 4.0–4.5 in the posterior/inferior segments) [41]. The LSI values were lower in the EGM-based approach compared with targeted LSI (*p* < 0.001); however, the rate of the atrial arrhythmias was comparable in both groups after 11.31 ± 1.70 months, with 90% in the EGM group and 91.7% in the LSI-based approach [41].

The use of EGM-based approaches for ablation strategies is currently limited to a small sample of clinical studies since these strategies are susceptible to artifacts, and interpreting unipolar signals in patients with atrial fibrillation can be challenging. Additionally, this approach has only been documented in patients with paroxysmal AF, thereby limiting its consideration to patients with persistent AF or prior ablations, where interpreting unipolar signals may be more challenging.

### 3.3 Contact Force and Force–Time Variables in Radiofrequency Ablation

Catheter–tissue interphase contact is a crucial variable in defining the success of a radiofrequency lesion creation [42]. Improved catheter contact enhances the interaction between the electrode surface and the myocardium, leading to a decrease in RF loss in the blood pool [42]. A higher CF correlates with a larger lesion size, thereby making it an important factor in successful ablations [43, 44].

More effective lesions are delivered by CFs within a range of 10 to 20 g, compared to lower forces of 2 g [4]. In turn, forces exceeding 40 g may proportionally create larger lesions but also increase the risk of tissue damage and steam pops [45, 46]. A CF between 10 and 22 g was found to be associated with the prevention of acute pulmonary vein reconnection, with a probability of over 95% (Fig. 2.5) [47].

Clinical studies have demonstrated the benefits of CF-guided ablations. In a study from Germany, the use of a CF compared to a no-CF ablation resulted in a reduced procedure time (128.4 ± 29 min vs. 157.7 ± 30.8 min, *p* = 0.001) and a significant reduction in the rate of arrhythmia recurrences after 12 months of follow-up (16.1% vs. 36.6%, *p* = 0.031) [20]. Similar outcomes were reported by Andrade *et al*. [48] in 2014, with a 12% arrhythmia recurrence rate in patients undergoing pulmonary vein isolation guided by CFs after 12 months of follow-up, compared to 34% in the non-CF group.

### 3.4 Force–Time Integral (FTI) as a Calculated Function

The introduction of CF ablation catheters has also allowed for the development of calculated indices that may estimate the extent of the lesion formation. The FTI is the product of multiplying the total RF time by the average CF and can be rapidly assessed during ablation. Both the FTI and average CF have been associated with transmural lesions [49]. Higher FTI values (>700 gs) correlate with 100% transmural RF lesions in the atrium [49].

The lessons learned from previous studies have contributed to the establishment of improved workflows during PVI. The EFFICAS II study (Optimization of Catheter Contact Force Improves Outcome of Pulmonary Vein Isolation for Paroxysmal Atrial Fibrillation) set ablation parameters for PVI using a CF target between 10 and 20 g and an FTI of 400 gs (Fig. 2.6). After 3 months of follow-up, 85% of the pulmonary veins remained isolated, and 15% of the reconnections correlated with areas where the catheters were unstable during ablation [21]. The continuity index, which can be used as a marker of stability and is based on the different positions of the catheter tip during ablation, was lower in areas without evidence of reconnection (4.1 ± 2.4), compared to the gap formation areas (8.4 ± 4.1) (*p* < 0.0001). Lesions with a continuity index <6 had a 98% chance of remaining isolated compared to 62% of those with a CI >6 (*p* < 0.0001) [21].

Previous findings from meta-analyses regarding the benefits of CF-guided ablations have been contradictory. Initial reports from observational studies showed a significant reduction in atrial arrhythmia recurrence, procedure time, and fluoroscopy time [50, 51]. However, these associations were less pronounced when considering only information from randomized control trials [51].

### 3.5 The Lesion Size Index (LSI)

The LSI is an automated module that has been integrated into different versions of the Ensite mapping system (Abbott Medical, Minneapolis, MN, USA), and considers multiple ablation parameters. It is obtained by integrating power, time, CF, and impedance data during RFA. The LSI was developed to understand the characteristics of *in vivo* lesions during AF and to predict the degree of myocardial damage [8, 52].

Animal studies have shown that the LSI correlates well with the FTI and lesion dimensions using different powers and a fixed CF with a parallel catheter orientation [52, 53]. Optimal LSI values that reach transmurality have been established as an LSI >4.0 in the posterior wall and >5.2 in other areas, to prevent the formation of conduction recovery (Fig. 2.7) [54]. Another study showed that patients who did not experience recurrence after 12 months of follow-up had possessed a higher average LSI during the first procedure [55]. Sundaram *et al*. [22] concluded that in their cohort, aiming for a minimum LSI of 6.0 in the left pulmonary veins ridge, 5.5 in the anterior, and 5.0 in the posterior segments of the pulmonary veins (PVs) resulted in an 86% freedom from atrial arrhythmias after two years of follow-up.

While the LSI offers promising results as an effective marker for lesion creation, it is currently only available in one manufacturer’s algorithm. Furthermore, the evidence supporting its use mainly originates from small observational studies [52, 53]. However, despite these limitations, the LSI offers valuable insights into lesion characteristics during AF ablation and may help to predict the likelihood of a successful lesion creation. Further research and larger studies are needed to establish its widespread clinical utility.

### 3.6 Ablation Index and Interlesion Distance in Radiofrequency Ablation

Advancements in CF, power, energy delivery parameters, catheter stability information (Fig. 2.8), and the understanding of lesion biophysics have led to the development of ablation indexes (AIs) that can objectively assess lesion creation and durability during RFA [56, 57]. Commercially available AIs, such as the Lesion Index (Abbott, Green Oaks, IL, USA) and the CARTO VISITAG™ Module (Biosense Webster, Irvine, CA, USA), integrate stability, CF, time, and power to aid in optimizing lesion formation.

Studies have shown that AIs are associated with significantly higher first-pass isolation rates, higher impedance drops, and lower atrial arrhythmia recurrence rates [56]. In patients with a prior PVI who underwent a second ablation independent of symptoms to assess lesion durability, their PV reconnection areas had lower AI and FTI values compared to non-reconnected segments [56]. An AI >370 in the posterior wall and 480 in the anterior roof areas correlated with no evidence of PV reconnection (Fig. 2.9) [56].

Other studies have suggested that an interlesion distance of ≤5 mm and a CF of >10 g are important factors for achieving acute durable lesions [58]. Hoffmann *et al*. [59] proposed aiming for an interlesion distance of 3–4 mm to increase the acute success rate of first-pass isolation (Fig. 2.10).

The CLOSE) protocol (Role of Interlesion Distance, Ablation Index, and Contact Force Variability) emphasizes the importance of contiguous lesions with an interlesion distance <6 mm and optimized RF lesions with an AI of at least 400 in the posterior wall and 550 in the anterior wall [23]. The CLOSE approach has demonstrated superiority in procedure time, RF time per PV circle, and incidence of adenosine-sensitive dormant conduction compared to conventional CF-guided approaches [23]. The freedom from atrial arrhythmias was higher in the CLOSE group after 12 months of follow-up (94% vs. 80%) [23].

A meta-analysis of the available studies comparing the use of AIs as the main strategy for ablation during PVI to other approaches showed favorable outcomes for the AI group, whereby AIs were associated with shorter procedure times, shorter ablation times, higher rates of first-pass isolation, less acute PV reconnections, and a lower incidence of atrial arrhythmias without a significant increase in complications [60].

### 3.7 Modification in Electrical Excitability Post-Ablation

Changes in the pacing threshold with the loss of capture in the atrial tissue after catheter ablation have been recognized as strong markers of transmural lesion creation. These changes can be used in addition to entrance and exit blocks to predict dormant areas of isolation [32, 61]

In patients undergoing PVI, the loss of pace capture at 10 mA/2 ms along the PVI line was associated with an entrance block in 95% of patients. In the remaining 5%, extra lesions were delivered to achieve the entrance block, and 50% of patients required additional ablation lesions to reach an exit block [62]. The loss of capture along the line after PVI is associated with a better outcome after 2 years of follow-up, and a higher success rate (83% vs. 52%) [24].

After circumferential ablation, high-output pace-capture identified a dormant conduction that required additional reinforcement lesions. The results of a study comparing high-output pacing at the ablation line, vs. adenosine, to recognize areas with persistent conduction after ablation showed a similar recurrence rate of 35% in both groups after a one-year follow-up [63].

These findings highlight the importance of monitoring changes in the pacing threshold and pace capture during catheter ablation procedures. They can serve as valuable tools to assess the effectiveness of lesion creation and predict the need for additional ablations to achieve complete isolation. However, longer-term follow-ups and larger studies are necessary to validate these findings and determine their broader clinical significance.

### 3.8 Imaging Techniques to Assess Ablation Effects

Various imaging techniques have been explored to improve catheter visualization, and stability, and reduce ionizing radiation during electrophysiology (EP) procedures.

Nowadays, intracardiac echocardiography (ICE) has become an essential tool in the practice of cardiac electrophysiology. Its introduction has allowed for a better understanding of cardiac anatomy, reduced fluoroscopy time—is an essential component of zero-fluoroscopy procedures—reduced the risks of complications, and improved patient outcomes after ablation [64, 65, 66, 67]. ICE permits real-time catheter visualization and confirms adequate catheter–tissue contact during ablation, thereby increasing the possibility of reaching transmurality more efficiently (shorter RF time, shorter procedure time, and more effective energy delivery) [68]. Local tissue changes on ICE that are indicative of an effective lesion creation include good catheter–tissue contact before ablation, swelling, tissue indentations or crater formation, and an increase in echogenicity; ICE can also predict the development of steam pops when accelerated bubbles are present in the catheter tip tissue interphase [64, 69].

Magnetic resonance imaging (MRI)-guided ablation has been studied as an alternative to visualize lesions in real-time, using T-2 sequences to assess for an edema and late gadolinium enhancement (LGE) series to predict necrosis. However, its implementation is currently limited to right-sided procedures, and more data are needed to establish its use in LA ablation or in the left ventricle [70, 71]. Challenges include the correlation between T-2 edemas and long-lasting lesions and the time required for LGE to be fully established, in addition to the EP laboratory device and equipment compatibility limitation, which remains a major area of concern [57, 71]. Additionally, studies have revealed that utilizing an MRI-guided, fibrosis-targeted ablation with PVI did not significantly improve ablation outcomes. This is thought to be secondary to the fact that the application of thermal injury to fibrotic tissue might not lead to the elimination of its arrhythmogenic potential [72].

In preclinical studies, this technique proved sensitive in identifying temperature changes in saline baths. However, despite its overall good performance, its sensitivity was found to be dependent on the distance between the antenna and the heat source [73]. An irrigated ablation catheter with microwave radiometry capacity was developed, and its ability to predict the development of steam pops was compared to conventional parameters, such as power, impedance, and catheter temperature in an animal model [74]; rate of increase in volumetric temperature (V temp) greater than 1.5 °C/s, as measured by microwave radiometry, emerged as the most powerful predictor of pop formation, outperforming prior conventional parameters in a multivariate analysis. Interestingly, no steam pops occurred when the V temp was maintained below 89 °C [74]. A similar catheter with the capability to adjust irrigation and the power to maintain a targeted tissue temperature exhibited wide but superficial lesions of 7–9.2 mm, and 4.3–5.5 mm, respectively [75].

Nicotinamide adenine dinucleotide (NADH), used in fluorometric imaging to assess catheter contact and predict lesion effectiveness, has shown promise in animals, although data from clinical studies are still pending [57, 76].

Near-field ultrasound (NFUS) imaging with transducers in the ablation catheter provides valuable feedback on catheter contact, lesion formation, and wall thickness [77, 78]. It can be useful in clinical applications for visualizing the electrode–tissue contact, measuring the wall thickness, identifying ablation lesions, and predicting lesion transmurality [77].

Optical coherence tomography (OCT) uses light to obtain high-resolution images and can precisely define ablation lesion characteristics [79]. Studies have shown that OCT can visualize ablation lesions with a power of ≥20 W and correlate lesion characteristics with histological findings [79].

Photoacoustic imaging may offer a real-time visualization of lesion progression and efficacy during ablation procedures. Although promising, this technique has not yet been proven in human studies [80].

The use of endoscopic laser ablation is currently under development. The concept consists of a 980 nm diode laser and a multi-lumen catheter with an inflatable balloon at the tip. Before ablation, the balloon is advanced into the LA, and inflated in the ostium of a PV. Additionally, the endoscope is introduced to guide ablation. The infrared laser can be aimed radially or at variable angles toward the catheter tip to create point-by-point circumferential lesions [81]. The first study in humans showed an acute PVI in 91% of patients, and freedom of atrial arrhythmias after a 12-month follow-up in 60% [82]. A second-generation device has been tested in human studies showing non-inferiority to RF energy after 12 months of follow-up (61.1% vs. 61.7%) [83], and to cryoablation (73% vs. 63%, *p* = 0.18) [84].

These imaging techniques have the potential to enhance the precision and success of catheter ablation procedures. However, further research and validation are needed before widespread clinical implementation, particularly to develop the practical and real-time utilization of such techniques.

## 4. Conclusions

Despite significant advancements in the anatomical approach for atrial fibrillation ablation over the past 25 years, achieving durable lesions without collateral damage remains a challenge in the field of cardiac electrophysiology. This challenge has driven the rapid evolution of our specialty, with a focus on improving lesion creation through the development of new energy delivery technologies.

To assess the effectiveness of lesions during ablation procedures, there are several surrogate tools available. Rather than relying on just one or two of these tools, it is recommended to use them in combination. This article proposes the consideration of the “10 commandments of lesion creation and assessment during AF ablation procedures” (Fig. 2), which can guide operators in their approach to durable lesions.

Understanding the biophysics of lesion creation and continuously improving lesion assessment techniques are essential for advancing catheter ablation of atrial fibrillation and achieving better long-term outcomes for patients. As the field continues to evolve, further research and innovation in lesion assessment will likely lead to even more effective and durable treatments for atrial fibrillation.
